# Near-infrared fluorescence imaging with indocyanine green for assessment of donor livers in a rat model of ischemia–reperfusion

**DOI:** 10.1186/s12876-022-02103-5

**Published:** 2022-01-20

**Authors:** Liyu Shan, Huan Chen, Lifei Yang, Zhe Feng, Yue Wang, Rongfeng Wang, Nana Zhang, Rongqian Wu, Yi Lv, Tao Ma

**Affiliations:** 1 National Local Joint Engineering Research Center for Precision Surgery and Regenerative Medicine, First Affiliated Hospital of Xi’an Jiaotong University, Xi’an, 710061 China; 2 Department of Hepatobiliary Surgery, The First Affiliated Hospital of Kunming Medical University, Kunming, 650032 China; 3 Department of Cardiovascular Surgery, First Affiliated Hospital of Xi’an Jiaotong University, Xi’an, 710061 China

**Keywords:** Indocyanine green, Near-infrared fluorescence imaging, Liver function assessment, Donor liver, Ischemia–reperfusion injury, Liver transplantation

## Abstract

**Background:**

Although marginal donor livers expand the donor pool, an ideal method for quantitatively evaluating the quality of donor livers has not been developed. This study aimed to explore the feasibility of indocyanine green (ICG) fluorescence imaging for estimating liver function in an ischemia–reperfusion model.

**Methods:**

Forty-eight rats were randomly and evenly divided into 8 groups: the control group and the experimental groups (I-VII). The portal vein blocking period was 0 min, 10 min, 20 min, 30 min, 40 min, 50 min and 60 min. After blood flow was reestablished and the hemodynamics stabilized, ICG was injected through the dorsal penile vein as a bolus, and the fluorescence signal was recorded for 30 min in real time. The fluorescence intensity (FI) curve of the liver was fitted with an asymptotic regression model. Fresh liver tissues and serum were obtained from the middle lobe of the liver on postoperative day (POD) 1 and POD 7 for histopathological evaluation and liver function tests.

**Results:**

The growth rate of the FI curve, parameter b3, decreased from groups I to VII. According to the two sudden changes in b3 (20 min, 50 min), the experimental groups could be classified into 3 groups (A, B and C). Hepatocytes in groups I-II showed slight edema, group III began to show obvious hepatocyte edema and vacuolar degeneration, and in groups VI-VII, severe hepatocyte degeneration, necrosis and large inflammatory cell infiltration were observed. Suzuki’s scores in the 3 groups were also significantly different (*P* < 0.01). At the same time, the serum liver function in the experimental groups showed a significant increase on POD 1 and a decrease on POD 7. The alanine aminotransferase (ALT), aspartate aminotransferase (AST), and total bilirubin (TB) levels of groups A, B, and C were significantly different on POD 1 (*P* < 0.05), and the ALT and direct bilirubin (DB) levels were significantly different on POD 7 (*P* < 0.05); the lactic dehydrogenase (LDH) level of the group C was significantly higher than that of the groups A and B on POD 1 and POD 7. Meanwhile, the 7-day survival rate of the rats in group C was poor compared to that of the rats in groups A and B (58.3% vs. 100% vs. 100%).

**Conclusion:**

ICG fluorescence imaging is effective for estimating the degree of liver damage and grading in an ischemia–reperfusion model. It probably has the potential for use in assessing the quality of the donor liver in liver transplantation.

## Background

The large demand for liver transplantation and the shortage of grafts are bottlenecks and challenges faced in the development of liver transplantation worldwide [1]. With the expansion of liver donor pools, traditional liver donor quality indicators cannot estimate donation after cardiac death (DCD) or marginal livers comprehensively and accurately [2, 3]. Because the subjective judgment of transplant doctors is limited by experience, pathology and imaging methods are invasive and lack convenience, a unified evaluation standard for liver donors has not yet been formed. It is of great clinical significance to find a noninvasive quantitative evaluation method.

Indocyanine green (ICG) is the only fluorescent imaging substance approved by the Food and Drug Administration (FDA) for use in humans [4]. After entering the human body, it can quickly bind to proteins and is selectively taken up by hepatocytes and excreted through the biliary tract as a prototype without hepato-intestinal circulation. ICG does not participate in biochemical reactions in the body and can be excited by near-infrared light at a wavelength of 760–810 nm, emitting fluorescence at 830 nm. Fluorescence imaging with ICG is widely used as an intraoperative navigation tool for various cancers [5–9], sentinel lymph nodes [10–14], and blood perfusion of tissues and organs [15–22]. As ICG is metabolized only by the liver, fluorescence imaging with ICG is expected to be ideal for assessing donor livers.

Hepatic ischemia–reperfusion injury (HIRI) refers to the reestablishment of blood flow after the liver endures ischemia for a certain period [23, 24], the inflammatory response increases, and even serious dysfunction occurs. HIRI is one of the main factors influencing the quality of donor livers, and it is closely related to the occurrence of nonanastomosed bile duct stenosis after liver transplantation [25–27]. In this study, we used ischemia time as a variable to investigate the feasibility and effectiveness of the ICG fluorescence imaging system for evaluating liver function in a rat liver ischemia–reperfusion model. We hope to lay the foundation for follow-up research on the assessment of donor livers in rat liver transplantation.

## Methods

### Experimental animals and groups

Forty-eight healthy male Sprague–Dawley (SD) rats weighing 280–320 g were provided by the Experimental Animal Center of Xi'an Jiaotong University. The rats were fed in the animal center, the temperature was maintained at 25 ± 2 °C, the humidity was controlled at 65–80%, 12 h of light/day were maintained, and the rats had free access to food and water. The animal protocol was designed to minimize pain to the rats in our experiment.

Animal feeding and management strictly followed the national Regulations on the Management of Experimental Animals and the Implementation Rules for the Management Conditions of Experimental Animals in Shaanxi Province. The experimental procedure was approved by the Animal Ethics Committee of Xi'an Jiaotong University and complied with the Declaration of Helsinki.

Before the experiment, all the rats were fasted for 8 h and allowed to drink freely.

**Fig. 1:**
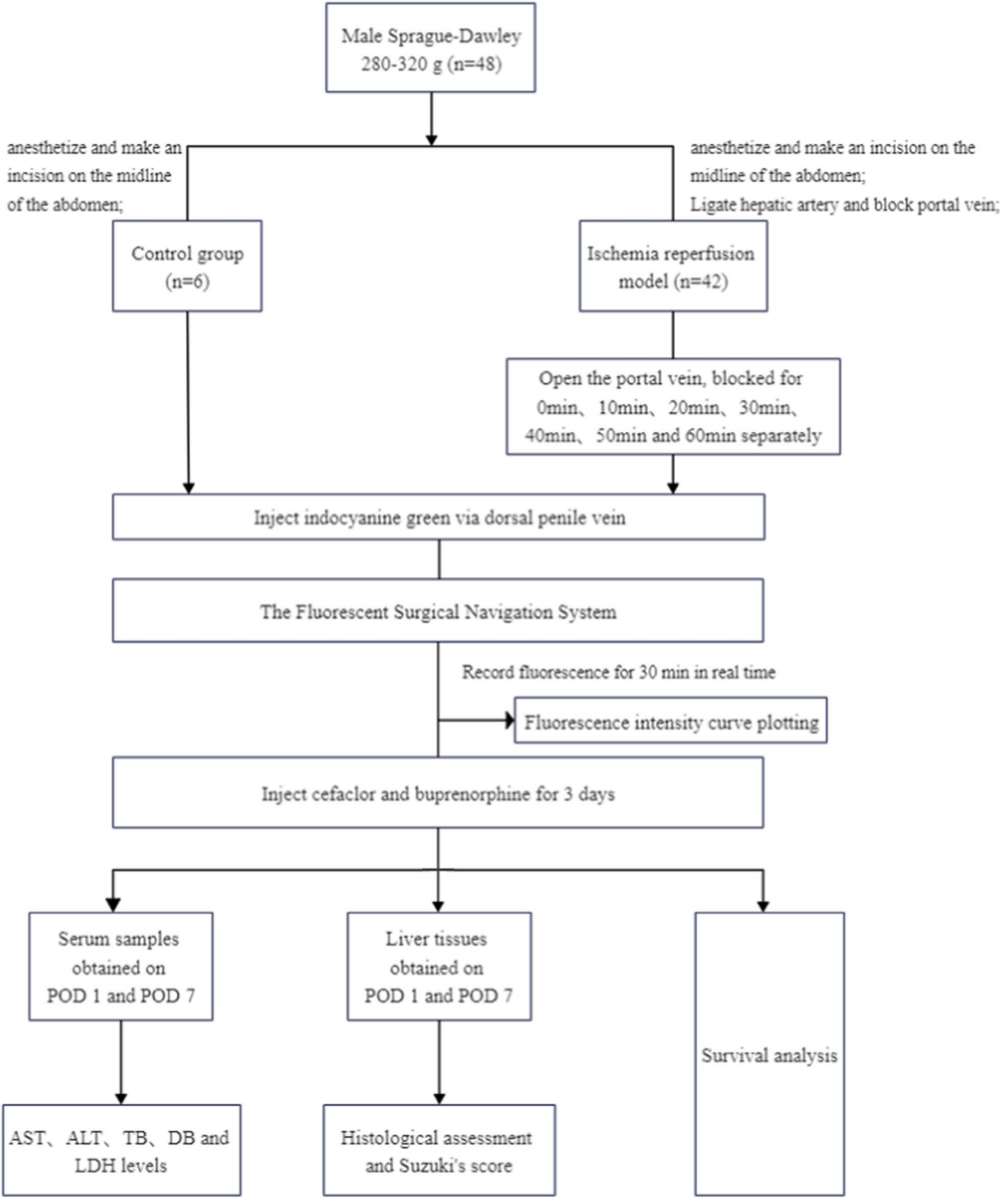
The experiment design

Forty-eight rats were randomly divided into 8 groups evenly. Ligation of the hepatic artery during the operation was performed in the experimental group rats but not in the control group rats. According to the blocking time of the portal vein, the rats in the experimental groups were divided into groups I-VII, with blocking times from 0 to 60 min. The experiment design is shown in Fig. 1.

### Equipment and reagents

The Open Fluorescent Surgical Navigation System (Magnetic Health care, Xi’an, China) and indocyanine green (25 mg, Dandong Yichuang Pharm., Dandong, China) were used. ICG was diluted in sterile water to yield a stock solution of 0.3 mg/ml for immediate use.

### Surgical procedure

**Fig. 2:**
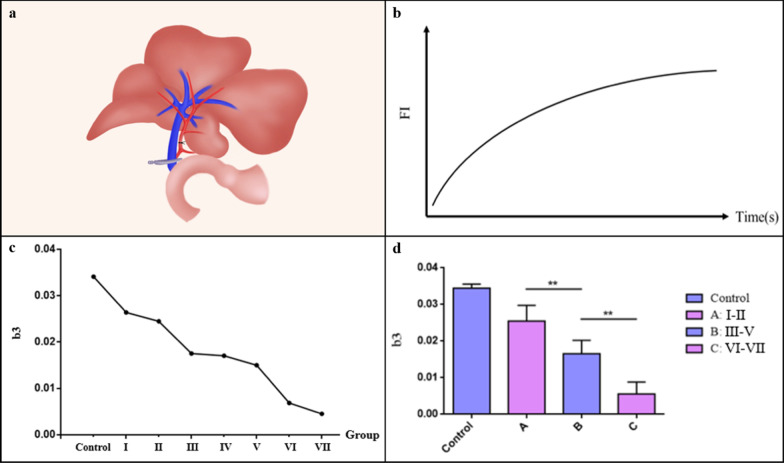
Surgical method and fluorescence curve analysis. **a** Method of establishing an ischemia–reperfusion model in rat liver. The hepatic artery was ligated with 6–0 silk sutures, and the portal vein was clamped with microvessel clips; **b** Fluorescence intensity curve. Fluorescence intensity first grew fast, then slowed, and finally reached a plateau phase; **c** We chose the asymptotic regression model to fit the fluorescence intensity curve. Parameter b3, which represents the growth rate of the FI curve, decreased from groups I-VII; **d** Group A included groups I and II, group B included groups III-V, and group C included groups VI-VIII. Parameter b3 was significantly different among groups A, B and C (*P* < 0.01)

In this experiment, the rats were anesthetized with 5% isoflurane inhalation. First, each rat was placed into the anesthesia induction box, and the flow valve was opened to the maximum for approximately 2 min. The rat was placed in the supine position after the respiratory rate decreased, the limbs were fixed, a mask was placed on the rat’s head, and the flow valve was adjusted to 0.6–0.8 L/min. All the abdominal skin was shaved and disinfected with povidone-iodine solution. An incision was made on the midline of the abdomen. After ensuring that there were no abnormalities of the abdomen and liver, the falciform ligament and connective tissues around the liver were dissected. The proper hepatic artery was freed from the upper edge of the upper duodenum and ligated with a 6–0 silk suture. Two milliliters of heparin saline (25 U/ml, 4 °C) was injected via the inferior vena cava to heparinize the whole body of the rat. The portal vein was moderately isolated and blocked with a clip close to the liver side. When the portal vein was blocked, a piece of gauze soaked with normal saline at 4 °C was gently placed to cover the surface of the rat's abdominal cavity to prevent water loss. The method for establishing the experimental groups is shown in Fig. 2a.

**Fig. 3:**
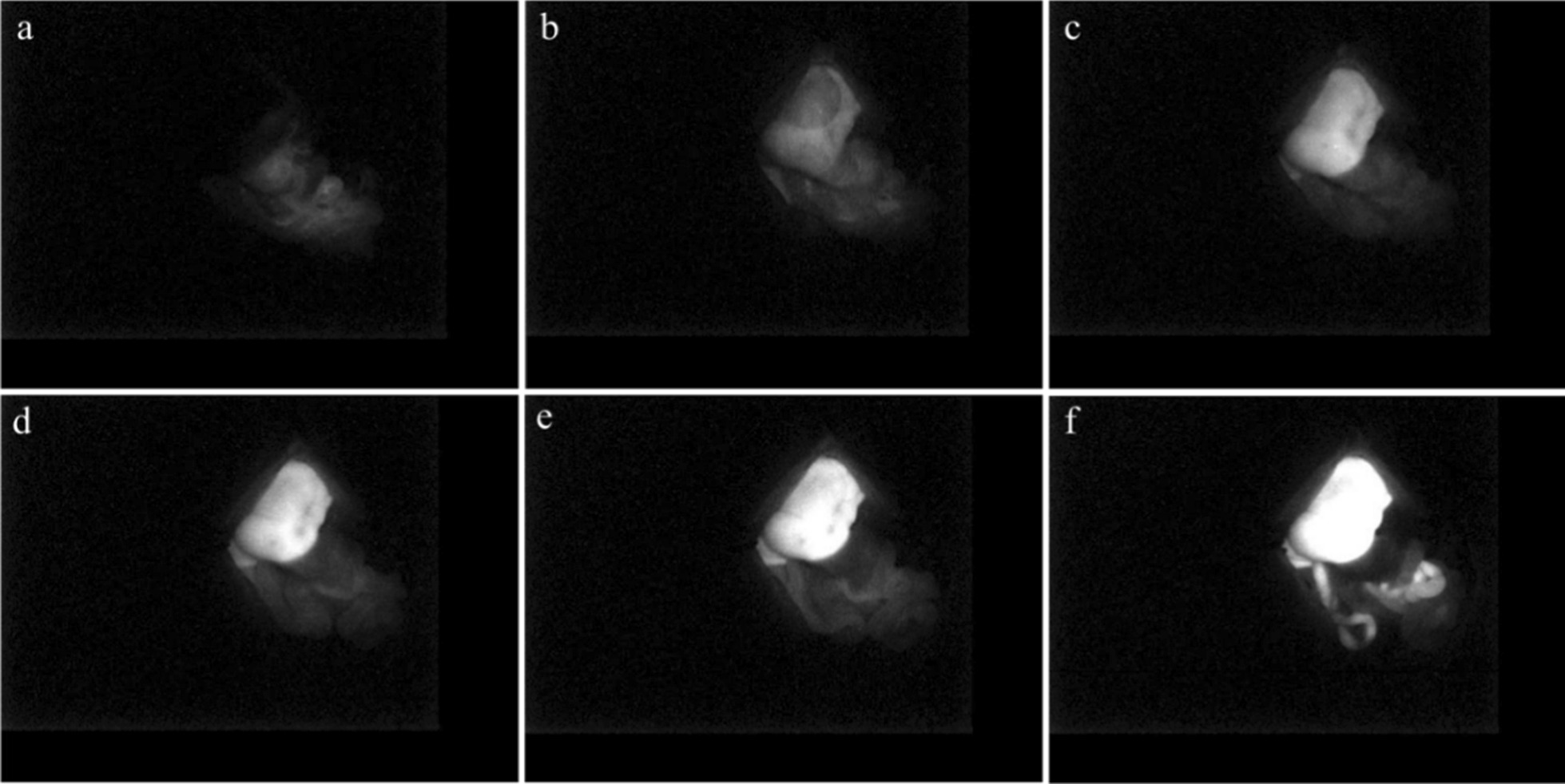
ICG fluorescence imaging process. **a** 5 s after ICG injection; **b** 15 s after ICG injection; **c** 5 min after ICG injection; **d** 10 min after ICG injection; **e** 20 min after ICG injection; **f** 30 min after ICG injection

After the occlusion times up, the clip was removed to open the portal vein. The ICG solution was then injected via the dorsal penile vein at 0.5 mg/kg. The injection time was set as the 0 point, and real-time recording of the rat liver fluorescence was carried out for 30 min. The process is presented in Fig. 3 with group III as an example. During the imaging process, the posture of the rat was kept constant, and normal saline was dropped on the surface of the organs to keep the abdominal organs moist. At the end of the recording, a dry cotton ball was used to absorb the excess water in the abdominal cavity. Nonabsorbable 3–0 silk sutures were used for two layers of consecutive stitches.

The rats were injected with cefaclor (16 mg/kg) and buprenorphine (0.05 mg/kg) for 3 days, subcutaneously on postoperative day (POD) 1, and intraperitoneally on POD 2 and POD 3.

### Sample collection

On POD 1 and POD 7, the rats in each group were sacrificed by obtaining blood samples through the abdominal aorta. The rat liver was washed with physiological saline at 4 °C and placed on dry gauze to absorb moisture. Liver tissues of approximately 1 cm in diameter were taken from the middle liver lobe and placed in a 4% paraformaldehyde solution for fixation. The serum was separated and stored at − 80 °C until analysis.

### Fluorescence intensity curve

Image J software was used to process and analyze the fluorescence imaging images of each group. The region of interest (ROI) was selected at the brightest position on the middle lobe of the rat liver, and the average pixel intensity in the ROI was obtained at different times. To accurately analyze the FI and changes, the initial fluorescence intensity was removed from the background fluorescence intensity. Finally, the FI curve was drawn, as shown in Fig. 2b. According to the trend of the curve, the appropriate model to fit was selected, and the various parameters were statistically analyzed.

### Assessment of liver function

Liver function was assessed by measuring the levels of alanine aminotransferase (ALT), aspartate aminotransferase (AST), direct bilirubin (DB), total bilirubin (TB) and lactic dehydrogenase (LDH). Serum samples were obtained on POD 1 and POD 7, and liver function was tested with an automatic biochemical analyzer (Chemray 800, Shenzhen, China).

### Histological evaluation of the liver

The specimens of each group were obtained on POD 1 and POD 7 and fixed with 4% paraformaldehyde. Paraffin embedding, hematoxylin and eosin staining, dehydration, transparency and mounting were carried out, and then microscopic examination was performed under an optical microscope. The samples were scored according to Suzuki’s standard [28, 29] to reflect the degree of liver damage as shown in Table 1.

**Table 1:** Suzuki’s histopathological scoring criteria

Score	Congestion (%)	Vacuolar degeneration (%)	Necrosis (%)
0	None	None	None
1	Very slight (10)	Very slight (10)	Single-cell necrosis
2	Slight (11–30)	Slight (11–30)	Slight (< 30)
3	Moderate (31–60)	Moderate (31–60)	Moderate (31–60)
4	Severe (> 60)	Severe (> 60)	Severe (> 60)

### Statistical methods

IBM SPSS Statistics 24.0 statistical software was used for analysis. Measurement data are expressed as the mean ± standard deviation ($\overline{x}$ ± SD), ANOVA and Student’s *t* test were used for comparisons between groups and within groups. *P* < 0.05 indicates that the difference is statistically significant.

## Results

### FI curve

The growth rate of the FI curve is fast at first, then slows down, and finally becomes stable. The fitting formula is as follows:

$$FI\left( t \right) = b1 - b2 \cdot e^{ - b3 \cdot t}$$

b1 is the upper limit of the FI curve, which represents the maximum fluorescence intensity; b2, an adjustment parameter, eliminates the influence of b1 on the "0 point", that is, b1–b2 is the initial FI; b3 represents the curve growth rate. b1 and b2 between groups were not significantly different (*P* > 0.05); b3 decreased between groups with longer portal vein blocking time, as shown in Fig. 2c. The b3 of groups II and V decreased sharply, and the corresponding portal vein block times were 20 min and 50 min, respectively. We then divided the groups into A, B, and C at 20 min and 50 min, as shown in Fig. 2d. The decrease in the b3 value between groups A, B, and C was significantly different (*P* < 0.01).

### Liver function evaluation

Compared with those of the control group, the ALT and AST levels of groups I-VII on POD 1 were significantly increased. The increase in groups I-II was smaller than that in groups III-V and VI-VII, and the increase in groups VI and VII was the largest, with a significant difference (*P* < 0.05); the levels of DB and LDH on POD 7 in groups VI and VII were significantly higher than those in groups I-V; the levels of DB and TB in group III were significantly higher on POD 1 than those in groups I-II.

With prolonged blocking time, the ALT and AST levels increased on POD 1 and obviously decreased on POD 7. The ALT and AST levels in groups VI and VII were still relatively high on POD 7, those in the other groups were maintained at a lower level.

The liver function indices, such as ALT and AST, of each group tended to be higher on POD 1 and lower on POD 7. There was no significant difference between the AST, TB, and LDH levels of groups I-V and the control group on POD 7, as shown in Table 2.

**Table 2:** Effect of different ischemia times on liver function (mean ± SD)

Liver function	Control	Group		POD 1	POD 7
ALT (U/L)	83.26 ± 10.31	A	I	116.28 ± 14.43	84.34 ± 5.58
			II	158.57 ± 26.48	73.22 ± 8.55
		B	III	267.64 ± 39.82^a^	56.27 ± 5.32^a^
			IV	345.47 ± 28.92^b^	74.08 ± 18.04^a^
			V	418.18 ± 17.31^b^	59.46 ± 3.68^a^
		C	VI	2038.33 ± 114.80^c^	90.51 ± 18.41^c^
			VII	2585.46 ± 99.56^d^	104.40 ± 8.00^c^
AST (U/L)	163.92 ± 40.72	A	I	348.49 ± 34.50	121.99 ± 10.10
			II	346.23 ± 36.88	129.85 ± 13.96
		B	III	587.63 ± 16.13^b^	114.34 ± 12.22
			IV	652.02 ± 34.83^b^	124.29 ± 9.84
			V	1000.94 ± 77.29^b^	140.45 ± 21.18
		C	VI	2163.72 ± 33.70^d^	222.83 ± 23.62^c^
			VII	2794.61 ± 177.41^d^	239.03 ± 44.12^c^
DB (µmol/L)	3.38 ± 0.92	A	I	3.40 ± 0.51	2.74 ± 0.50
			II	4.01 ± 0.20	2.82 ± 0.15
		B	III	4.88 ± 0.52	3.28 ± 0.11^a^
			IV	4.91 ± 0.08	3.33 ± 0.15^a^
			V	4.96 ± 0.35	3.62 ± 0.06^b^
		C	VI	4.92 ± 0.25	4.14 ± 0.24^c^
			VII	5.35 ± 0.63	4.99 ± 0.03^d^
TB (µmol/L)	9.51 ± 0.29	A	I	8.43 ± 1.20	8.43 ± 1.20
			II	6.98 ± 0.03	7.02 ± 0.12
		B	III	10.75 ± 0.44^b^	7.73 ± 0.24
			IV	9.13 ± 1.00^b^	9.26 ± 0.23^b^
			V	8.51 ± 0.29^b^	9.47 ± 0.44
		C	VI	11.44 ± 0.21^d^	10.79 ± 0.92^c^
			VII	12.50 ± 0.19^d^	12.06 ± 0.35^c^
LDH (U/L)	733.18 ± 102.0	A	I	1421.22 ± 276.70	590.93 ± 14.68
			II	1452.79 ± 324.34	736.05 ± 146.78
		B	III	2341.30 ± 105.82	939.20 ± 43.17
			IV	1471.21 ± 81.76	725.76 ± 154.91
			V	1549.70 ± 163.10	953.03 ± 78.40
		C	VI	1562.79 ± 55.89	1889.12 ± 96.55^d^
			VII	1938.16 ± 58.62^d^	2392.23 ± 206.01^d^

^a^*P* < 0.05

^b^*P* < 0.01 versus group A (I–II)

^c^*P* < 0.05

^d^*P* < 0.01 versus group B (III–V)

### Histological evaluation of liver

**Fig. 4:**
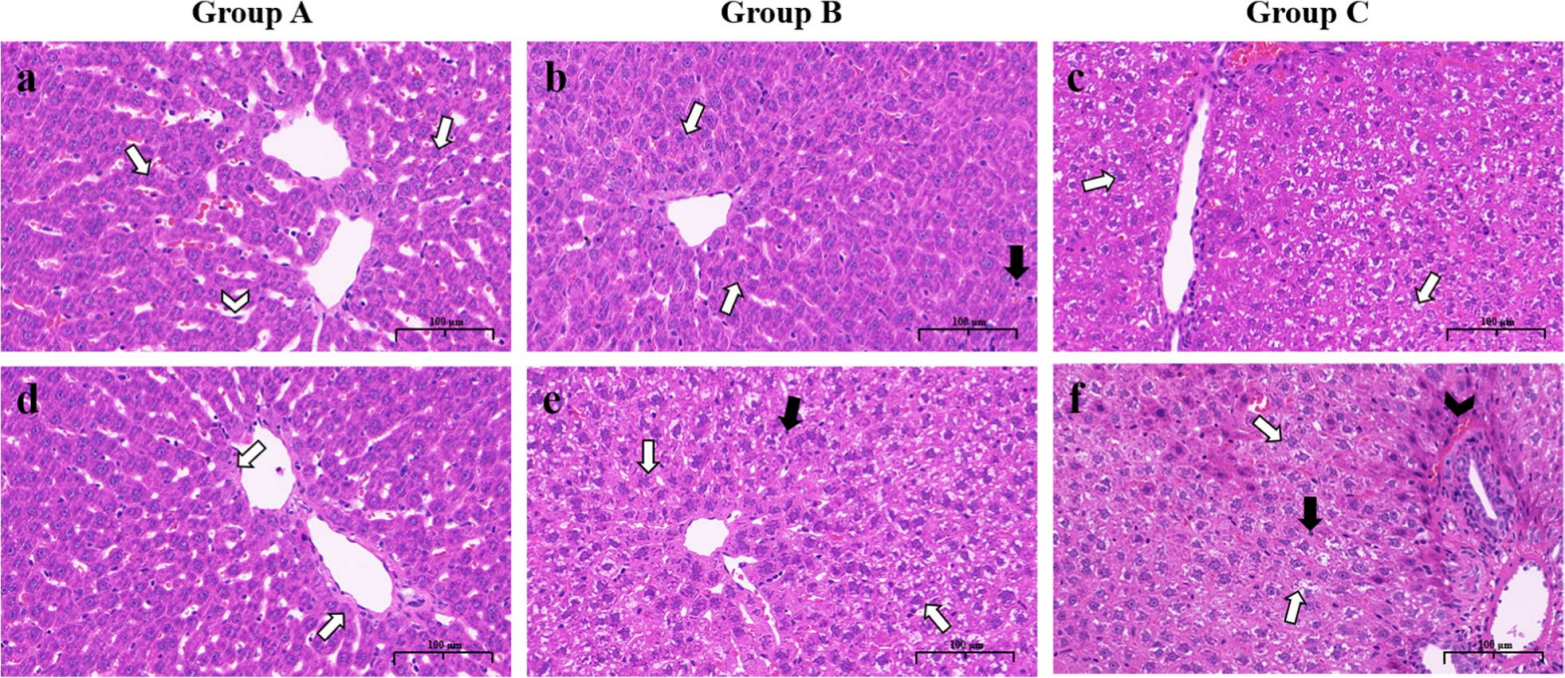
Histological examination of the liver on POD 1 and POD 7 among groups A, B and C; **a**–**c** Liver tissue on POD 1 in groups A-C; **d**–**e** Liver tissue on POD 7 in groups A-C

As shown in Fig. 4a and d, the hepatocytes (white arrow) in groups I and II had mild edema, the hepatocyte cords were arranged and separated normally by normal hepatic sinusoids (white chevron), and the nuclei were round, large, and centered, without inflammatory cell infiltration. In Fig. 4b, hepatocyte edema (white arrow) occurred in group III on POD 1, and vacuolar degeneration and apoptotic bodies (black arrow) were observed. The degree of hepatocyte edema (white arrow) in Fig. 4e was higher in groups IV and V. The hepatic sinusoids appeared narrow, and the range of vacuolar degeneration and apoptotic bodies (black arrow) was larger on POD 7. As shown in Fig. 4c, the hepatocytes (white arrow) had severe edema, and large areas of hepatocyte degeneration and necrosis were seen on POD 1 in group VII. Besides, necrosis was obvious (white arrow), and large areas of inflammatory cell infiltration (black chevron) were seen on POD 7 in Fig. 4f.

**Fig. 5:**
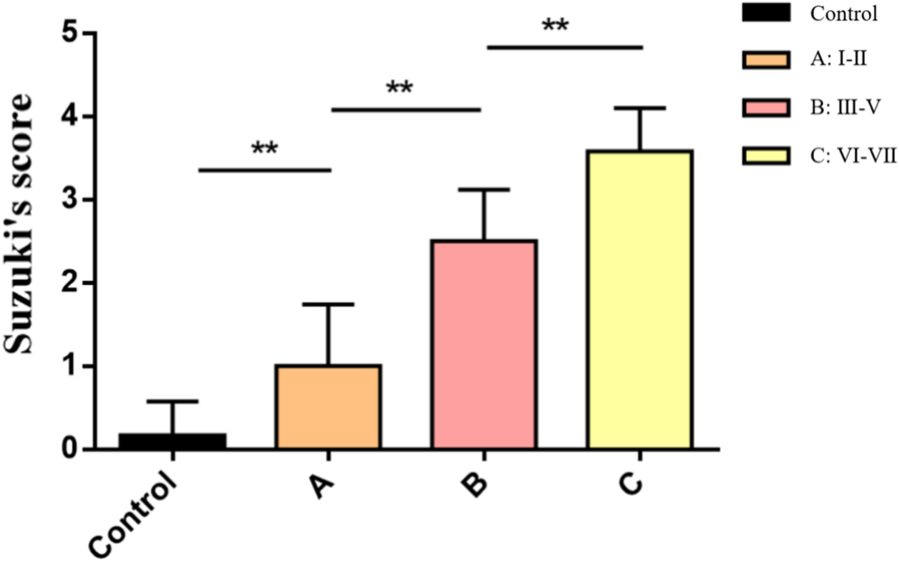
Suzuki’s scores among groups A, B and C were significantly different on POD 1. (*P* < 0.01)

Suzuki’s pathological scores were significantly different among the three groups: group C was higher than group A and B, and group A had the lowest score (*P* < 0.01) as shown in Fig. 5.

### Survival

**Fig. 6:**
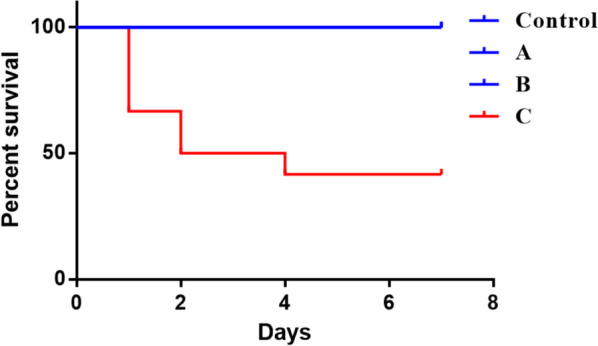
The survival rate among the groups from POD 1 to POD 7. Seven rats in group C died in 7 days, and 4 of them died on POD 1. No death happened in the other groups

The survival curve of each group was analyzed. The rats in the control and I-V experimental groups survived well, and there was no death at 7 days. Seven rats in groups VI and VII died within 7 days after the operation, and 4 of them died on POD 1. The survival curve is shown in Fig. 6.

## Discussion

Since ICG received FDA certification in 1959, it has been widely used as a dye for clinical positioning and visualization, such as in the biliary tract [30–34], in the ureter [35–37], and in hepatocellular carcinoma (HCC) [8, 9, 38–40]. With the deeper development of ICG fluorescence imaging technology, it gradually showed the potential for use in the quantitative assessment of graft function.

In 2010, C. Hoffmann [41] used ICG fluorescence imaging during kidney transplantation and found perfusion defects that are invisible to the naked eye; in 2015, Yoshikuni Kawaguchi [42] found portal vein thrombosis not detected by Doppler ultrasound during liver transplantation, revealing the potential of ICG fluorescence imaging technology for improving the survival rate of transplantation. Fluorescence imaging quantitative assessment of overall liver function was first reported in Narasaki's research [43] and later improved by Damien Dousse [22].

Damien Dousse [22] proposed an exponential model but only observed and measured it for 3 min. The data used for analysis was the first 150 s. No data were excluded in our experiment. After we analyzed our data, we obtained a model consistent with Damien Dousse, but we used the data from the first 30 min for analysis. Their research suggested that the growth rate of the FI curve is faster in patients undergoing primary nonfunction (PNF) after transplantation and retransplantation. Our conclusions in the ischemia–reperfusion injury model are opposed to theirs. The reason may be as follows: In their study, the experiment was carried out in human liver transplantation, while we developed a rat model of ischemia–reperfusion injury. They had 4 patients with PNF after transplantation and 6 patients with retransplantation. The sample was small, and the quality of the graft was uncontrollable due to the complexity during the surgical operation. However, our research was carried out in animal experiments, the liver ischemia time was controllable, and the sample size was larger.

The FI curve was characterized by 3 phases: an ascending phase, a gentle phase, and a plateau phase, which generally corresponded to the process of ICG metabolism. Perfusion through sinusoids is then taken up, transported, and excreted by hepatocytes to reach equilibrium. We hypothesize that with prolonged ischemic time of the liver, reperfusion injury is more severe, and the ability of hepatocytes to take up ICG is impaired, so the b3 value decreased. The transport and excretion capacity of liver cells may have been damaged accordingly, and the specific mechanism needs further research.

In this study, we found that 20 min and 50 min of ischemia time were related to two sudden changes in parameter b3. Groups A, B, and C, which were established by these two sudden changes, showed significant differences in serum liver function and histopathology, and the morphological recovery of cells occurred later than serological indicators. We found that the mortality in group C (41.7%) was much higher than that in the other groups (0%). In addition to the damage to liver function, part of the reason may be attributed to bowel congestion and infarction. This is understandable because, in the process of using ischemia–reperfusion as a representative factor, we must make the surgical approach as close as possible to the rat liver transplantation model to increase the reliability of the results. Other tests in our study also provide evidence that ICG fluorescence imaging has the potential to be used to quantitatively assess the quality of donor livers in different aspects.

Many previous studies [44–46] have concluded that liver transplantation in rats within 20 min of warm ischemia can achieve a better survival rate. After approximately 45 min of warm ischemia, the incidence of biliary obstruction and mortality increases after transplantation. These pieces of evidence prove that b3 may be used to classify liver quality.

The only difference between the control group and group I was whether the hepatic artery was ligated. Although the hepatic artery provides a smaller blood flow to the liver than the portal vein, it contains a large amount of oxygen, which provides for liver metabolism. In the FI curve, the b3 of the two groups was significantly different, but it was not seen in other inspection methods in this experiment. This suggests that ICG fluorescence imaging for detecting liver hypoxic-ischemic damage may be more sensitive than other testing methods.

Of course, this experiment takes ischemia–reperfusion injury as a representative factor as a preliminary study for the fluorescence assessment of the quality of the donor liver in liver transplantation, and we will carry out further experiments in the rat liver transplantation model. In this experiment, hepatic artery ligation was performed in the experimental groups to be consistent with the operation situation of the recipients in rat liver transplantation where hepatic artery ligation was required.

## Conclusion

The quantitative evaluation of liver function by ICG fluorescence imaging is feasible and effective in the ischemia–reperfusion model of rat liver, and it has the potential to be used in donor liver evaluation in a rat liver transplantation model in future studies.

## Data Availability

The datasets generated and/or analyzed in the experiment are available from the corresponding author on reasonable request.

## Abbreviations

ICG: Indocyanine green
FI: Fluorescence intensity
POD: Postoperative day
ALT: Alanine aminotransferase
AST: Aspartate aminotransferase
TB: Total bilirubin
DB: Direct bilirubin
LDH: Lactic dehydrogenase
DCD: Donation after cardiac death
FDA: Food and drug administration
HIRI: Hepatic ischemia–reperfusion injury
SD: Sprague–Dawley
ROI: Region of interest
